# Is Verapamil Effective for Treating Patients With Chronic Rhinosinusitis With Nasal Polyps? A Systematic Review

**DOI:** 10.1111/coa.70041

**Published:** 2025-10-15

**Authors:** Fangxing Yin, Haris Ali, Alaa Abdelhalim, Hassan A. Elhassan

**Affiliations:** ^1^ School of Medicine and Dentistry Queen Mary University of London London UK; ^2^ Medway Hospital Kent UK; ^3^ Department of ENT Homerton University Hospital Foundation Trust London UK

**Keywords:** chronic rhinosinusitis, nasal polyposis, novel therapy, P‐gP, type 2 inflammation, verapamil

## Abstract

**Objective:**

Identify and summarise the evidence on the efficacy of low‐dose verapamil use for adult patients with Chronic Rhinosinusitis with Nasal Polyps (CRSwNP).

**Methods:**

Two reviewers independently searched the data sources until the 28th of December 2023. From 262 studies identified, PRISMA guidelines were implemented to include three randomised controlled trials that used verapamil for patients diagnosed with CRSwNP. Primary endpoints were extracted, including quality‐of‐life questionnaires and imaging gradings.

**Results:**

Across 116 participants, all three studies showed statistically significant improvement in the subjective scores of patients who experienced symptoms in the verapamil group. The mean differences between the two intervention groups and placebo in SNOT‐22 were −19.17 (95% CI: −30.76 to −7.58) and −27.7 (95% CI: −49.36 to −6.05). On the objective metrics, statistically significant improvement in TNPS or LMS between groups was seen in two out of the three studies (0.01 [*p* = 0.001], −5.2 [*p* = 0.02]).

**Conclusion:**

Although verapamil usage resulted in significant improvements in the subjective and objective measurements of the severity of CRSwNP, the current evidence is insufficient to support widespread usage of verapamil for CRSwNP. Further studies should focus on recruiting a clinically significant number of patients, while ensuring robustness and homogeneity in the methodological design.

**Summary**

- Chronic rhinosinusitis with nasal polyps (CRSwNP) is associated with P‐glycoprotein (P‐gP) overexpression, and verapamil has shown potential in reducing Th2 inflammation by inhibiting p‐gP‐mediated cytokine secretion.
- Significant improvements were observed in subjective symptom severity measures such as SNOT‐22 and TNSS, indicating verapamil's potential for symptom relief.
- Objective outcome measures, including CT and endoscopic scores, showed inconsistent results, possibly due to short study durations and small sample sizes.
- Limitations of the studies included small cohorts, differences in methodology, and potential biases in outcome assessment and reporting.
- Further research with larger cohorts, standardized protocols, longer follow‐up, and better endotyping is needed to establish verapamil's clinical efficacy and safety in CRSwNP management.

## 1 Introduction

Chronic rhinosinusitis (CRS) is a common ENT pathology that affects > 10% of the adult population in Europe and the United States [1]. The economic burden is substantial, with direct healthcare costs in the United States alone estimated at $10 billion annually, compounded by indirect costs such as lost productivity due to work absences [2]. Mild CRS symptoms are managed by saline irrigation and intranasal corticosteroids (INS) according to the European Position Paper on Rhinosinusitis and Nasal Polyps (EPOS) 2020 guideline [3]. An incomplete understanding of CRS pathophysiology, limited molecular differentiation of its endotypes and lack of effective targeted therapies leave many patients grappling with chronic, relapsing symptoms [3, 4]. These challenges significantly impact patients' quality of life (QoL) and pose ongoing difficulties for clinicians. The need for novel therapy is pressing.

Chronic rhinosinusitis with nasal polyps (CRSwNP) constitutes up to 80% of type 2 endotype CRS, which affects around 25%–40% of CRS patients [3, 4]. CRSwNP is characterised by polypoid mucosa and increased type 2 inflammation. The inflammation is linked to the overexpression of a membrane efflux pump in the sinonasal epithelial membrane named P‐glycoprotein (P‐gP). P‐gP regulates the secretion of T‐helper 2 (TH2) polarising cytokines, leading to an uncontrolled eosinophilic immune response [5]. Verapamil hydrochloride (HCl), a non‐dihydropyridine calcium channel blocker as well as a first‐generation inhibitor of P‐gP, has been studied as a potential drug to treat CRSwNP when used at a low dose. By inhibiting P‐gP, verapamil is capable of blocking many inflammatory mediating cytokines that contribute to the formation of nasal polyps (NP), including IL5, IL6 and thymic stromal lymphopoietin (TSLP), ultimately leading to TH2 production [6, 7]. There have been several trials investigating the use of verapamil for CRSwNP. The objective of this review is to provide a summary of the current available evidence on the efficacy of low‐dose verapamil use for CRSwNP.

## 2 Materials and Methods

### 2.1 Protocol Registration and Guideline

The protocol for this review was registered on PROSPERO (CRD42024507732). The Preferred Reporting Items for Systematic Review and Meta‐Analyses (PRISMA) guidelines were used as a basis to conduct a systematic review of the available literature [8]. The PICO framework was implemented. The population is adult patients who suffer from CRSwNP, the intervention is low‐dose oral verapamil HCL. Comparison is made against placebo, no treatment or the current standard of care, and the outcome is assessed using quantifiable results such as Sino‐Nasal Outcome Test‐22 (SNOT‐22), CT score (Lund‐Mackay score) and endoscopic findings (Lund‐Kennedy score).

### 2.2 Search Strategy

PubMed, Embase, Ovid MEDLINE, Cochrane Database and Google Scholar were searched up until the 28th of December, 2023 to identify studies. Two domains were used to search and were connected with ‘AND’; whereas the individual terms in the domain are combined with ‘OR’. The search terms used for the first term include: ‘Verapamil’, ‘Verapamil hydrochloride’, ‘Verapamil HCL’, ‘52‐53‐9’, ‘Calan’, ‘Isoptin’. The second search terms were ‘Chronic rhinosinusitis’, ‘Chronic rhinosinusitis with nasal polyposis’, ‘Chronic rhinosinusitis Type 2’, ‘CRS’, ‘CRSwNP’, ‘Nasal polyps’, ‘Nasal polyposis’. Google Scholar was searched to incorporate grey literature to complement the breadth of results, the term ‘Verapamil for chronic rhinosinusitis’ was used due to the differences in the search term function. Two reviewers (F.Y. and H.A.) conducted the literature search independently.

Studies that were not related to our objective were identified through their title and abstract and excluded in the initial screening stage. Full‐text versions of studies were obtained where the abstracts were insufficient to determine their significance. We were able to retrieve the full texts of all relevant studies.

#### 2.2.1 Selection Criteria

We included studies that compare verapamil to placebo and corticosteroids, which is the current treatment of CRSwNP. Studies that use verapamil monotherapy and combination therapy are both included, irrespective of the route of administration. Experimental studies that used nasal polyp explants were excluded. Other endotypes of CRS besides diffuse type 2 inflammation CRS with nasal polyps are not included due to differences in pathophysiology. Studies and articles other than randomised controlled trials were not used as there is no quantifiable data.

### 2.3 Data Extraction

Three studies were selected, and the data extraction process was completed by two reviewers (F.Y. and H.A.) independently. Discrepancies were resolved through discussion. We followed Cochrane guidance [9] and extracted the following methodological endpoints from the studies: study type, number of patients treated in the verapamil group and control group, the control group treatment, patient demographics (age and gender), dosage, treatment period, outcomes and respective results. The adverse effects of verapamil are also documented where reported by the studies.

## 3 Results and Analysis

### 3.1 Search and Selection

**FIGURE 1:**
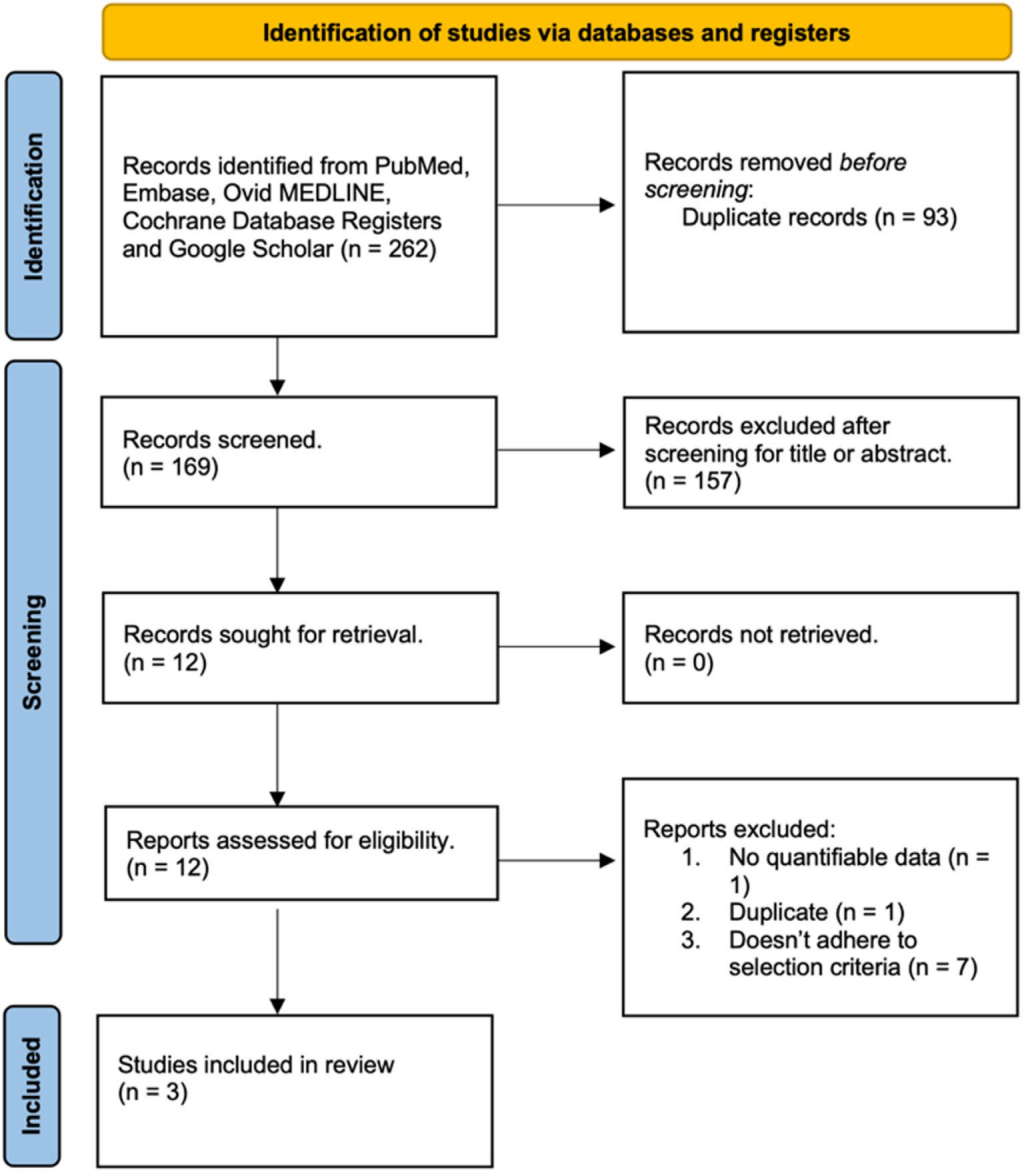
Flow chart of study selection.

Our search identified 262 studies from the databases. After screening titles and abstracts, we eliminated duplicates and studies incompatible with our PICO criteria. Upon evaluating the full texts and applying our selection criteria to the 12 potential studies, 9 further studies were excluded for the reasons mentioned in Figure 1.

Three studies were selected in the end, and the extracted data is summarised in Table 1 [6, 10, 11]. It is worth noting El Salam et al. is ‘grey literature’ identified through Google Scholar.

**TABLE 1:** Characteristics of selected studies.

Study	Study design	Treatment (no. of pts)	Control (no. of pts)	Male: female ratio	Age (mean and standard deviation)	Age range	BMI	Treatment group	Dosage and frequency	Control group treatment	Outcomes	Treatment period (months)
Miyake et al. (2017)	Double blind RCT	10	10	Treatment: 60%/40% Control: 60%/40%	48.8 ± 12.8	18–80	Means (SD) Treatment: 26.3 (2.6) Control: 30.5 (6.9)	Oral verapamil HCL	80 mg, TID	Placebo	SNOT‐22 score, VAS score, LMS and LKS	2
Nabavi et al. (2022)	Double blind RCT	18	18	Treatment: 50%/50% Control: 66.7%/33.3%	38.9 ± 7.0	Treatment: 29–51 Control: 22–49	Median (IQR) Treatment 25.65 (5.25) Control: 37.50 (8.00)	Irrigation + INS + Oral verapamil HCL	80 mg, TID	Irrigation + INS + Placebo	SNOT‐22 score and LMS	3
El Salam et al. (2022)	RCT	30	30	Treatment: 80%/20% Control: 76.7%/23.3%	38.55 ± 5.91	Treatment: 30–50 Control: 34–50	N/A	Topical verapamil	2 puffs in each nostril, BID	Topical Beclomethasone Dipropionate Monohydrate (2 puffs in each nostril BID)	TNSS and TNPS	3

Abbreviations: INS = Intranasal corticosteroids, LKS = Lund‐Kennedy Score, LMS = Lund‐McKay Score, pts = patients, RCT = randomised controlled trial, SNOT‐22 = Sino‐Nasal Outcomes Test‐22, VAS = visual analogue scale.

### 3.2 Participation and Randomisation

The three studies included 116 adult patients, all of whom were diagnosed with CRSwNP and presented to their respective clinic/hospital. The common exclusion criteria are patients with comorbidities such as gastrointestinal hypomotility and heart failure, or patients taking drugs such as aspirin and beta blockers due to potential contraindications or interactions with verapamil. All participants were then randomly divided into treatment and control groups for Miyake et al. and Nabavi et al. No significant difference was noted between the two groups of patients in terms of disease severity before intervention. However, El Salam et al. did not explicitly state the method used for the process of allocation. Statistical analysis of the baseline characteristics showed the TNSS was significantly different between the two groups (*p* = 0.047) raising some concerns of allocation bias.

All selected studies were prospective randomised controlled trials, with Nabavi et al. and Miyake et al. being double‐blinded. El Salam et al. did not provide information on blinding. In terms of the control group, El Salam et al. also differ as they compare topical verapamil with topical corticosteroid instead of placebo.

**FIGURE 2:**
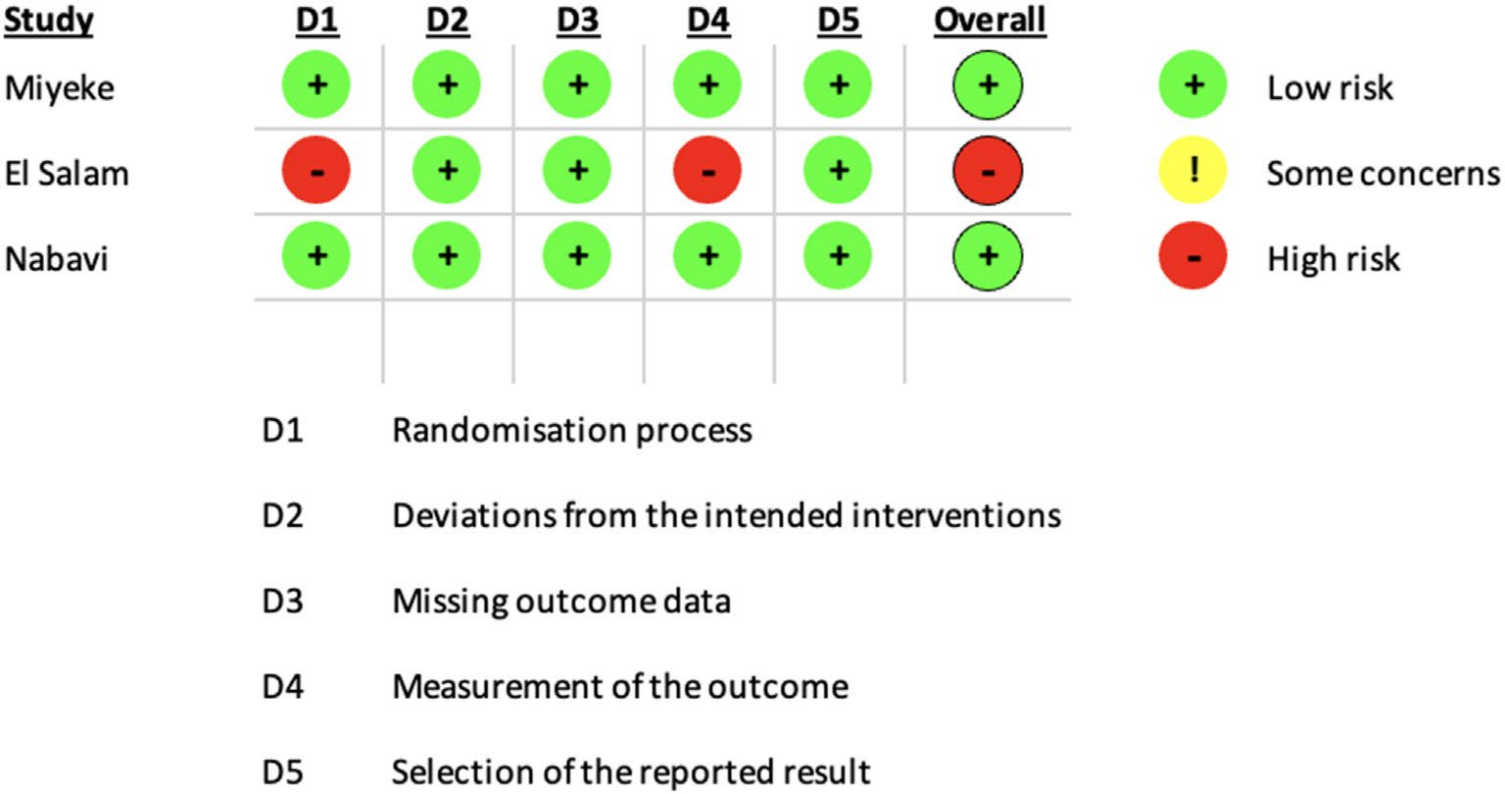
The risk assessment of the studies using RoB2.

Risk bias assessment was conducted using version 2 of the Cochrane risk‐of‐bias tool for randomised trials (RoB2) [12], assessing five domains in each study. This was done independently by F.Y. and H.A. with common ground reached after discussion. The result can be seen in Figure 2. The overall risk of bias is low for Miyake et al. and Nabavi et al., and high for El Salam according to the algorithm.

### 3.3 Results

#### 3.3.1 Synthesis Methods

Given the different outcome tests, we performed subgroup analysis. SNOT‐22 is a subjective questionnaire that assesses the severity based on the patient's symptoms across 22 items on a scale of 0 (no symptom at all) to 5 (worst possible symptom) [13]. Similarly, TNSS assesses six domains on a scale of 0 (no symptoms) to 6 (severe symptoms). LKS and TNPS are both secondary endpoints based on endoscopic findings, and LMS is based on CT findings. These scores were all measured at the start of the trial as a baseline, and at the end of the treatment period. The outcomes were categorised into two subgroups for our statistical analysis. The mean differences between the treatment and control group were calculated for each study, and the final standardised mean difference (SMD) was calculated using Cohen's D. Cambridge effect size calculator was used for data calculation and SPSS was used for data synthesis and presentation [14].

#### 3.3.2 Outcomes

Oral verapamil HCL demonstrated its ability to reduce the severity of SNOT‐22 scores from ‘severe’ (Nabavi et al.: 53.44 ± 14.61; Miyake et al.: 55.9 ± 31.5) to ‘moderate’ (Nabavi et al.: 29.88 ± 18.8; Miyake et al.: 27.3 ± 29.19) in the treatment group. After calculations, there is a statistically significant reduction in the final mean differences between the treatment group and the placebo group for both Nabavi et al. (−19.17 [95% CI: −30.76 to −7.58, *p* = 0.001]) and Miyake et al. (−27.7 [95% CI: −49.36 to −6.05, *p* = 0.01]). The raw mean differences alongside the P value for each outcome are shown in Table 2. Note that the mean difference shown is between the baseline and the end of the treatment period.

**TABLE 2:** The calculated mean differences of the three studies between the treatment and the control group, at the end of their respective treatment period.

Study	Outcome	Mean difference (95% CI)	*p*
Nabavi et al. (2022)	SNOT‐22	−19.17 (−30.76, −7.58)	0.001
	LMS	0.45 (−3.47, 4.37)	0.784
Miyake et al. (2017)	SNOT‐22	−27.7 (−49.36, −6.05)	0.01
	LMS	−5.2 (−9.58, −0.82)	0.02
	VAS	−37.97 (−60.01, −15.93)	0.001
	LKS	−1.05 (−2.88, 0.77)	0.25

		Verapamil	Corticosteroids	Mean difference (95% CI)	*p*
El Salam et al. (2022)
	TNSS	−2.72 (−2.89, −2.55)	−3.38 (−3.69, −3.07)	0.66 (0.53, 0.79)	0.001
TNPS	−1.48 (−1.81, −1.15)	−1.47 (−1.91, −1.03)	0.01 (−0.19, 0.21)	0.001

For El Salam et al., the mean difference is 0.66 (95% CI: 0.53, 0.79, *p* = 0.001). There is a significant reduction in the TNSS score for both groups and the difference between topical verapamil and topical corticosteroids is minimal in terms of patient‐reported symptoms.

For the objective measures, there is variation in the mean differences across the three studies. The final mean difference in LKS that Miyake et al. showed at −1.05 (95% CI: −2.88, 0.77, *p* = 0.25) is not significant at week 8. In El Salam et al., the final mean difference for TNPS between groups is 0.01 (*p* = 0.001), with both groups producing similar improvement in polyp score after management. For LMS, Miyake et al. found that the final mean difference is −5.2 (95% CI: −9.66 to −0.74; *p* = 0.02), demonstrating a significant reduction in the CT score favouring the verapamil group. However, this is not the case for Nabavi, which showed that the final mean difference in LMS is 0.45 (*p* = 0.784), not considered statistically significant.

**FIGURE 3:**
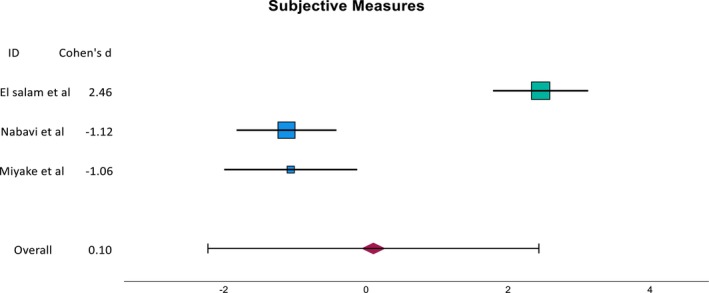
The SMD of the subjective outcome of the corresponding study.

**FIGURE 4:**
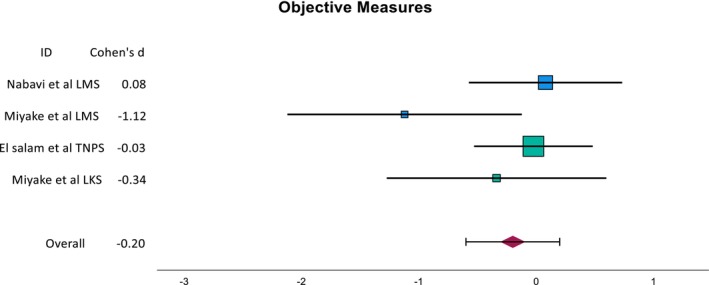
The SMD of the objective outcome of the corresponding study.

The calculated SMD of the subgroups are presented as seen in Figures 3 and 4. The SMD for SNOT‐22 and TNSS is −1.12 (95% CI: −1.8, −0.4) and −1.05 (95% CI: −2.03, −0.06) for Nabavi et al. and Miyake et al., respectively. The overall SMD is skewed by the large SMD of the TNSS result from El Salam et al. This is due to a small pooled standard deviation likely caused by the small sample size as well as the nature of data distribution.

Small overall improvements were observed in the objective measures, with a combined standardised mean difference (SMD) of −0.20. However, the actual value may be even lower due to the influence of the TNPS result from El Salam et al., where a corticosteroid group was used as the control. Across the studies, objective measures generally improved where verapamil was used, except for the LMS result from Nabavi et al.

## 4 Discussion

All the studies showed a statistically significant improvement in the subjective measure of patient symptom severity, demonstrating that verapamil both orally and topically seems to be effective in relieving the patient's symptoms, and at a similar level to topical steroids, according to El Salam et al. However, results were not so clear‐cut in the objective metrics, as for the endoscopic findings only El Salam et al. reported significant improvement in TNPS. An intermediate level of overall LMS improvement was seen with Nabavi et al. not producing statistically significant results compared to their control group. The lack of improvement in the objective scale might be caused by the short time frame between the baseline and the end of treatment, as it takes longer for the damaged/fibrosed mucosa to show improvements radiologically. Moreover, all the studies reported that low‐dose verapamil was easily tolerated by patients and no safety concerns were raised which is promising for future studies.

These three studies provide further clinical relevance to previously proposed theories. The correlation of P‐gP overexpression and the pathogenesis of CRSwNP is backed by clinical studies and laboratory findings. A higher concentration of P‐gP is seen in CRS patients, particularly in CRSwNP patients who suffer from eosinophilic TH2 polarised inflammation [15]. Furthermore, higher P‐gP secretion levels are linked with increased disease severity as discerned by subjective and objective indicators. Studies have shown that P‐gP not only acts as a biomarker for endotyping and prognosis, but also participates in epithelial cell‐driven inflammation. In vivo experiments found rapid intracellular P‐gP uptake carried out by mucus exosomes with mucociliary flow [16]. The recipient cells were found to have a significantly increased P‐gP activity. This is linked to the locally dysregulated inflammatory response found in the sinonasal epithelium [17]. Found in the epithelial cells in the upper and lower respiratory tract, P‐gP is an ATP‐dependent efflux protein associated with excreting many inflammatory cytokines including IL‐5, IL‐6 and TSLP, promoting TH2 inflammation [18]. By inhibiting P‐gP, verapamil reduces epithelial cell secretion of these TH2 polarising cytokines, providing some control over local inflammation and ultimately alleviating symptoms as supported by subjective measures. P‐gP also contributes to mometasone resistance and, by inhibiting P‐gP, verapamil can enhance mometasone retention as seen in Nabavi et al. [19, 20]. This effect has already been seen in in vitro studies, but it is promising to see continuing in vivo in these studies [21, 22, 23].

Verapamil, widely used for cardioactive effects (e.g., angina, hypertension, SVT) and as first‐line prophylaxis for cluster headaches, is generally well‐tolerated at doses of 240 to 320 mg per day in healthy individuals, with the minimum dose of 240 mg per day appearing free of side effects [10]. However, it has adverse effects like abdominal pain, dizziness, tachycardia, erectile dysfunction and rare but serious risks such as gingival hyperplasia, paraesthesia, atrioventricular block and hypotension [24]. In the reviewed studies, all three applied exclusion criteria and minimal dosing to mitigate risks, with Miyake et al. and El Salam et al. reporting no significant side effects between the treatment and control groups (*p* > 0.05). Despite this, Miyake et al. terminated their study early after 20 patients due to underdosing concerns at 80 mg thrice daily, suggesting low‐dose verapamil may be insufficient for patients with higher P‐gp overexpression, potentially requiring higher doses. If verapamil's efficacy does not outweigh its risks, its applicable population may be limited, though its safety profile in cluster headaches and the lack of significant side effects in these studies suggest it may be a reasonable candidate for further clinical trials in chronic rhinosinusitis.

The studies had limitations: small sample sizes (a priori power analysis indicated 79 patients per group needed for minimal clinical difference) [25], methodological heterogeneity (Nabavi et al. used verapamil as combination therapy with INS, potentially causing synergistic effects; El Salam et al. used topical verapamil, affecting efficacy and bioavailability) and outcome measurement biases. TNSS and SNOT22, being subjective, are prone to recall bias, though likely equal across groups with double‐blinding. El Salam et al. lacked clarity on TNPS blinding, risking observer bias and did not respond to inquiries. Miyake et al. and Nabavi et al. used independent observers for LKS and LMS. Miyake et al. had two placebo dropouts, using last‐observation‐carried‐forward for missing data, unlike the other studies. Only Miyake et al. disclosed a conflict of interest, with co‐author Blier holding a P‐gp inhibition patent for chronic rhinosinusitis, raising bias concerns.

These factors mentioned above have made our meta‐analysis less robust. Due to the limited number of studies investigating verapamil in CRSwNP, we pooled results from oral and topical administration to provide a preliminary synthesis of available evidence. Both routes target the same pathophysiological mechanism (calcium channel blockade), which may justify their combined analysis despite potential differences in bioavailability and local effects. As demonstrated by the results, there was a lack of a significant difference between oral and topical routes, as evidenced by overlapping confidence intervals, suggesting that pooling these data provides a preliminary estimate of verapamil's overall effect in CRSwNP, though we recognise the need for future studies to explore route‐specific outcomes.

There is no strong evidence favouring verapamil as monotherapy or combination therapy; neither was there strong evidence that suggests one route of administration was better than the other, as seen by the confidence intervals overlapping in the forest plot. In addition, a positive correlation was found between the discounted efficacy and patients having a higher BMI, as demonstrated by the linear regression analysis (*p* = 0.01), with an estimated range of 18.5–34.1 (calculated as mean ± 3 SD), spanning from normal to obese.

The overall quality of evidence is low as the included studies were of variable quality, notably caused by small sample size and the heterogeneity in administration routes and data collection methods, which restricts the generalisability of our findings. Emerging evidence highlights a link between P‐gp overexpression and Th2‐associated inflammation in CRSwNP; however, with clinical research on verapamil for CRSwNP still in its infancy, recommending its widespread use in clinical practice at this stage would be unwise. Balancing the risk of side effects of verapamil and its benefits will be a vital consideration for future studies. Other implications include recruiting a clinically sufficient number of patients with a variety of demographics to achieve adequate power and relevance to the wider population. As mentioned earlier as well, the short duration of treatment and follow‐up may have limited the impact on measurable outcomes and future studies may want to extend these. Better endotyping such as measuring mucus P‐gP will help link verapamil to a therapeutic target, and ensuring robustness and homogeneity in the methodological design will lead to stronger evidence for efficacy and indication. We recognise the limited number of eligible RCTs reduces the robustness of our meta‐analysis. However, as the first systematic review of verapamil for CRSwNP, this work consolidates the available evidence, identifies methodological shortcomings and guides the design of future high‐quality trials.

## 5 Conclusion

Chronic rhinosinusitis with nasal polyps is a complex and difficult‐to‐manage condition. Verapamil, highlighted in EPOS 2020 [3] as a therapy of interest for its role in P‐glycoprotein inhibition, shows promise as a low‐cost option with fewer long‐term side effects than biologics or systemic corticosteroids. Our review provides the first systematic synthesis of clinical data on verapamil for CRSwNP. While preliminary findings suggest potential benefit, the evidence is limited by small sample sizes, methodological heterogeneity and variable study quality. Verapamil cannot yet be recommended for clinical use, but its biologic plausibility and safety profile justify further well‐powered, methodologically robust RCTs.

## Author Contributions

F.Y. conceived and designed the experiments. F.Y. and H.A. performed the experiments. F.Y. and H.A. analysed the data. F.Y. and H.A. wrote the manuscript. A.A. reviewed and edited the manuscript. H.A.E. supervised the project. All authors reviewed and approved the final manuscript.

## Ethics Statement

The authors have nothing to report.

## Conflicts of Interest

The authors declare no conflicts of interest.

## Data Availability

The data that support the findings of this study are available from the corresponding author upon reasonable request.
